# Generation and characterisation of two D2A1 mammary cancer sublines to model spontaneous and experimental metastasis in a syngeneic BALB/c host

**DOI:** 10.1242/dmm.031740

**Published:** 2018-01-01

**Authors:** Ute Jungwirth, Antoinette van Weverwijk, Miriam J. Melake, Ann F. Chambers, Qiong Gao, Marc Fivaz, Clare M. Isacke

**Affiliations:** 1 The Breast Cancer Now Toby Robins Research Centre, The Institute of Cancer Research, 237 Fulham Road, London, SW3 6JB, UK; 2 German Cancer Research Center, DKFZ, 69120, Heidelberg, Germany; 3 Department of Oncology, University of Western Ontario, London, Ontario, N6A 4L6, Canada

**Keywords:** Mammary cancer, Metastatic sublines, Syngeneic, Spontaneous metastasis, D2A1, BALB/c

## Abstract

Studying the complex mechanisms underlying breast cancer metastasis and therapy response necessitates relevant *in vivo* models, particularly syngeneic models with an intact immune system. Two syngeneic spontaneously metastatic sublines, D2A1-m1 and D2A1-m2, were generated from the poorly metastasising BALB/c-derived D2A1 cell line by serial *in vivo* passaging. *In vivo* and *in vitro* analyses revealed distinct and shared characteristics of the metastatic D2A1-m1 and D2A1-m2 sublines. In particular, D2A1-m1 cells are more aggressive in experimental metastasis assays, while D2A1-m2 cells are more efficient at disseminating from the primary tumour in spontaneous metastasis assays. Surprisingly, classical metastasis-associated *in vitro* phenotypes, such as enhanced proliferation, migration and invasion, are reduced in the sublines compared to the parental cell line. Further, evasion of immune control cannot fully explain their enhanced metastatic properties. By contrast, both sublines show increased resistance to apoptosis when cultured in non-adherent conditions and, for the D2A1-m2 subline, increased 3D tumour spheroid growth. Moreover, the enhanced spontaneous metastatic phenotype of the D2A1-m2 subline is associated with an increased ability to recruit an activated tumour stroma. The metastatic D2A1-m1 and D2A1-m2 cell lines provide additional syngeneic models for investigating the different steps of the metastatic cascade and thereby represent valuable tools for breast cancer researchers. Finally, this study highlights that morphology and cell behaviour in 2D cell-based assays cannot be used as a reliable predictor of metastatic behaviour *in vivo*.

## Abstract (precis)

**Summary:** We describe two D2A1 mouse mammary cancer sublines with enhanced spontaneous metastasis in a syngeneic host, and highlight the limitations of *in vitro* assays to predict *in vivo* metastatic behaviour.

## INTRODUCTION

To study the complex mechanisms of breast cancer metastasis and therapy response in advanced disease, it is important to have relevant *in vivo* models. Ideally, the model recapitulates the full metastatic cascade, including growth of a primary tumour, dissemination of tumour cells into the circulation, colonisation of secondary sites and the development of macrometastatic disease. In addition, to assess the impact of the immune system, an immunocompetent syngeneic model is required. A recent study has molecularly characterised 12 mouse mammary cancer cell lines and performed phenotypic analysis of the primary tumours grown in syngeneic hosts (Yang et al., 2017). To date, the best characterised spontaneous breast cancer metastasis model is the BALB/c-derived 4T1 cell line (Aslakson and Miller, 1992) and the 4T1 sublines selected for increased metastasis to the bone and lung (Lelekakis et al., 1999; Tester et al., 2000) or brain (Lockman et al., 2010). More recently, Johnstone and colleagues have derived and characterised a spontaneously metastasising variant of the C57BL/6-derived murine medullary mammary adenocarcinoma cell line E0771 (Johnstone et al., 2015), allowing for metastasis studies to be performed in an alternative mouse strain. However, there is still an increasing demand for independent models both for study validation and to address the inter- and intratumour heterogeneity of human disease.

In this study, we describe the generation of two breast cancer cell sublines, D2A1-m1 and D2A1-m2, derived from parental D2A1 cells. The parental D2A1 cell line was derived from a mouse mammary tumour in a BALB/c mouse implanted with the transplantable D2 hyperplastic alveolar nodule cell line (Mahoney et al., 1985; Miller et al., 1989; Morris et al., 1993). In a recent comprehensive analysis of 12 mouse mammary cancer cell lines (Yang et al., 2017), D2A1 cells are classified as oestrogen receptor (ER)- and ErbB2/HER2-negative, *Pik3ca* and *Trp53* wild type, having a ‘claudin-low’ transcriptional profile and assignment to the luminal B subtype. *In vivo*, D2A1 tumours have a spindle-cell histology with ∼18% Ki67 (Mki67)-positive cells and ∼20% caspase 3-positive nuclei. It was originally reported that 4 weeks after orthotopic inoculation into immunocompromised nude BALB/c mice, two of five mice showed visible macrometastatic disease in the lungs, with the remaining mice showing lung micrometastatic disease upon histological examination (Morris et al., 1993). Subsequently, it was reported that parental D2A1 cells can colonise the lungs of immunocompetent BALB/c mice if injected via the tail vein in an experimental metastasis assay (Shibue and Weinberg, 2009), and that rates of spontaneous metastasis in BALB/c can be increased by pre-irradiation of the mammary gland (Bouchard et al., 2013), albeit still with a relatively low metastatic burden.

The D2A1-m1 and D2A1-m2 sublines were derived by serial inoculation of tumour cells in BALB/c mice followed by recovery from the lung tissue *ex vivo*. Characterisation of these two metastatic sublines demonstrates them to have both overlapping and distinct phenotypes in *in vivo* assays, *in vitro* assays and by gene expression profiling. In particular, the D2A1-m1 subline displays an enhanced ability to colonise the lungs and other tissues in experimental metastasis assays, whereas the D2A1-m2 subline shows a robust and reproducible ability to colonise the lungs in a spontaneous metastasis assay (inoculation into the mammary fat pad), associated with an increased ability to recruit an activated tumour stroma. Consequently, these two D2A1 sublines provide useful and complementary models to interrogate the different stages of the metastatic cascade.

## RESULTS

### Generation of spontaneously metastatic D2A1 sublines

**Fig. 1.**
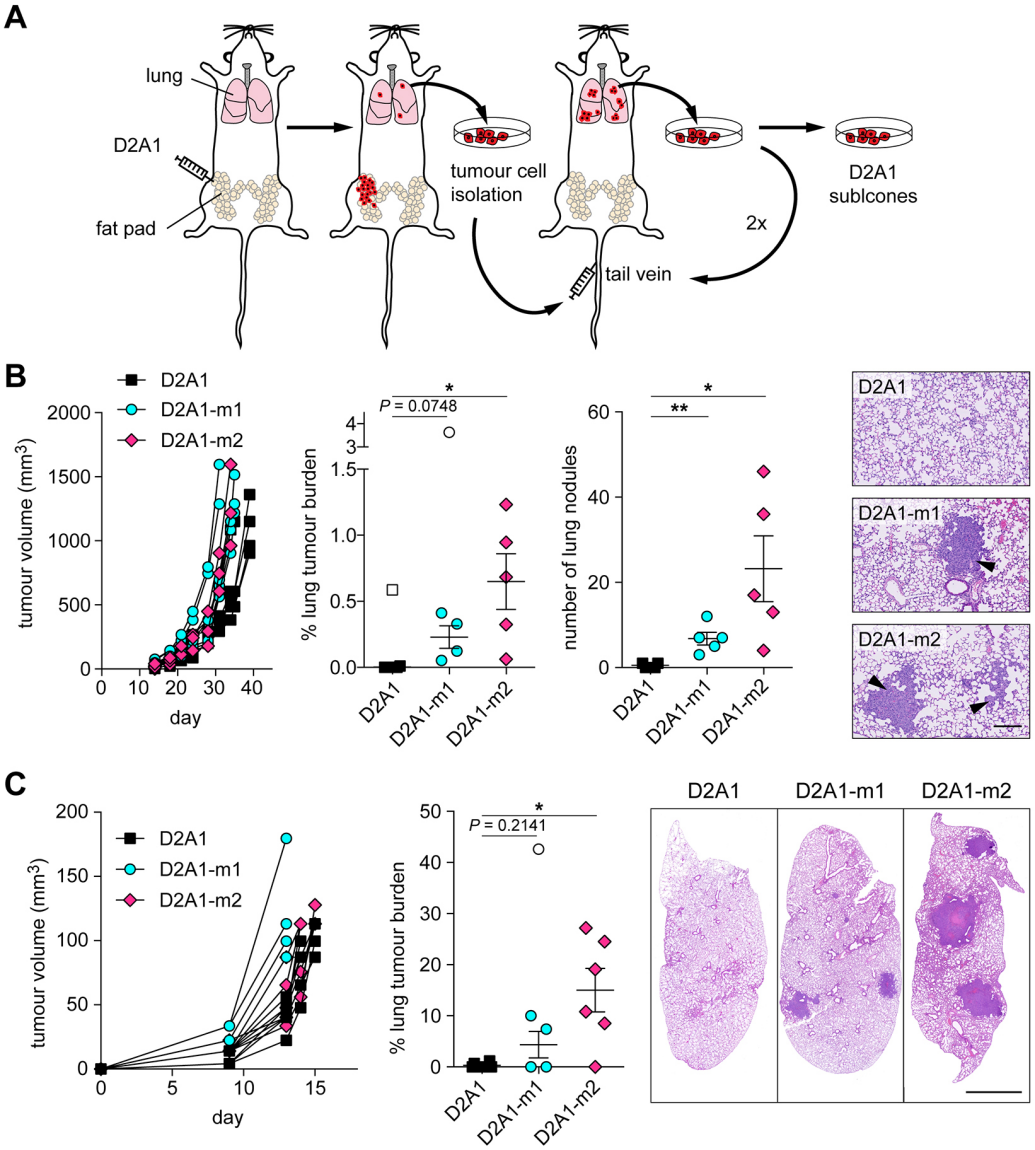
**Generation of syngeneic spontaneously metastatic D2A1 sublines.** (A) Diagram outlining the strategy for selection of the metastatic sublines (see Materials and Methods). (B,C) 5×10^4^ D2A1, D2A1-m1 or D2A1-m2 cells were inoculated into the fourth mammary fat pad of BALB/c mice (*n*=5-6 mice per group). Growth of tumours in individual mice is shown. (B) Mice were culled between days 31-39, when the primary tumours reached a diameter of 12-14 mm (left). Metastatic burden in the lungs was monitored by % tumour area and number of lung nodules per lung section. Significant outliers are shown as white symbols. Data are mean values per mouse ±s.e.m. Representative images of lung metastases (arrowheads) are shown (right). Scale bar: 200 µm. (C) Primary tumours were surgically excised when they reached ∼6 mm in diameter (day 13-15). All mice were culled on day 43 when the first mouse showed signs of ill health. Data are mean values per mouse ±s.e.m. for primary tumour growth and metastatic burden in the lungs as assessed by % tumour area per lung section. Significant outliers are shown as white symbols. Representative images of lung sections are shown (right). Scale bar: 2 mm. *P<0.05; **P<0.01.

The scheme for the generation of the D2A1 sublines is shown in Fig. 1A. The two sublines were derived independently. In brief, for each subline, parental D2A1 cells were inoculated orthotopically into the fourth mammary fat pad of an immunocompetent BALB/c mouse. When the primary tumour reached 10-12 mm in diameter, the lungs were harvested individually from each mouse at necropsy, dissociated, and placed into culture. Tumour cells that grew out were expanded and inoculated into the tail vein of a recipient mouse and 11-13 days later, lungs were removed at necropsy. In total, three rounds of intravenous inoculation were performed, resulting in the selection of the independent metastatic sublines, D2A1-m1 and D2A1-m2.

In an initial experiment to assess the metastatic competency of the selected sublines, parental D2A1, D2A1-m1 and D2A1-m2 cells were inoculated orthotopically into the mammary fat pad of recipient BALB/c mice. Animals were culled individually between days 31 and 39 when the primary tumours reached 12-14 mm in diameter (Fig. 1B). Consistent with the published literature (Bouchard et al., 2013; Morris et al., 1993; Yang et al., 2017), parental D2A1 cells readily formed primary tumours, but, with the exception of a single large metastatic nodule in one mouse, gave rise to only small metastatic nodules in the lungs, with the majority of mice having undetectable metastatic disease. All mice inoculated with D2A1-m1 and D2A1-m2 cells had metastatic disease as monitored both by tumour area and number of metastatic nodules, with the D2A1-m2 subline giving rise to the highest metastatic burden in this spontaneous metastasis assay. Although there was a difference in the number of metastatic nodules in mice inoculated with the D2A1-m1 and D2A1-m2 sublines, and an increase in the percentage tumour area in the lungs, the average size of the lung nodules was similar. Of note, although the D2A1-m1 and D2A1-m2 primary tumours grew slightly faster than the parental D2A1 tumours, in subsequent experiments no differences in primary tumour growth were observed (see below).

To better mimic the clinical setting in breast cancer, spontaneous metastasis assays in BALB/c mice were then performed in which the primary tumour was surgically resected at a small size (∼6 mm in diameter; day 11-13) and all mice were culled on day 43 (Fig. 1C). Despite the increased length of time of the experiment, when lungs were examined at autopsy, there was only limited metastatic disease in the parental D2A1 inoculated mice, whereas the D2A1-m2 subline gave rise to extensive tumour burden in the lungs. The D2A1-m1 subline gave mixed results, with two of five mice showing no metastatic disease and the remaining three mice having readily detectable macrometastatic nodules. The enhanced metastatic burden observed in mice in which the primary tumour was resected (Fig. 1C) likely reflects two factors. First, in experiments in which the primary tumour was not removed, mice were culled when the primary tumour reached its maximum allowable size (31-39 days) (Fig. 1B). By contrast, following tumour resection, the study was conducted over a longer time period (43 days), allowing increased growth of metastatic deposits (Francia et al., 2011). Second, in experimental mouse models, there have been numerous reports that removal of a primary tumour can enhance growth of metastatic lesions (O'Reilly et al., 1994). In addition, these observations suggest that dissemination of cells from the primary tumour is an early event.

**Fig. 2.**
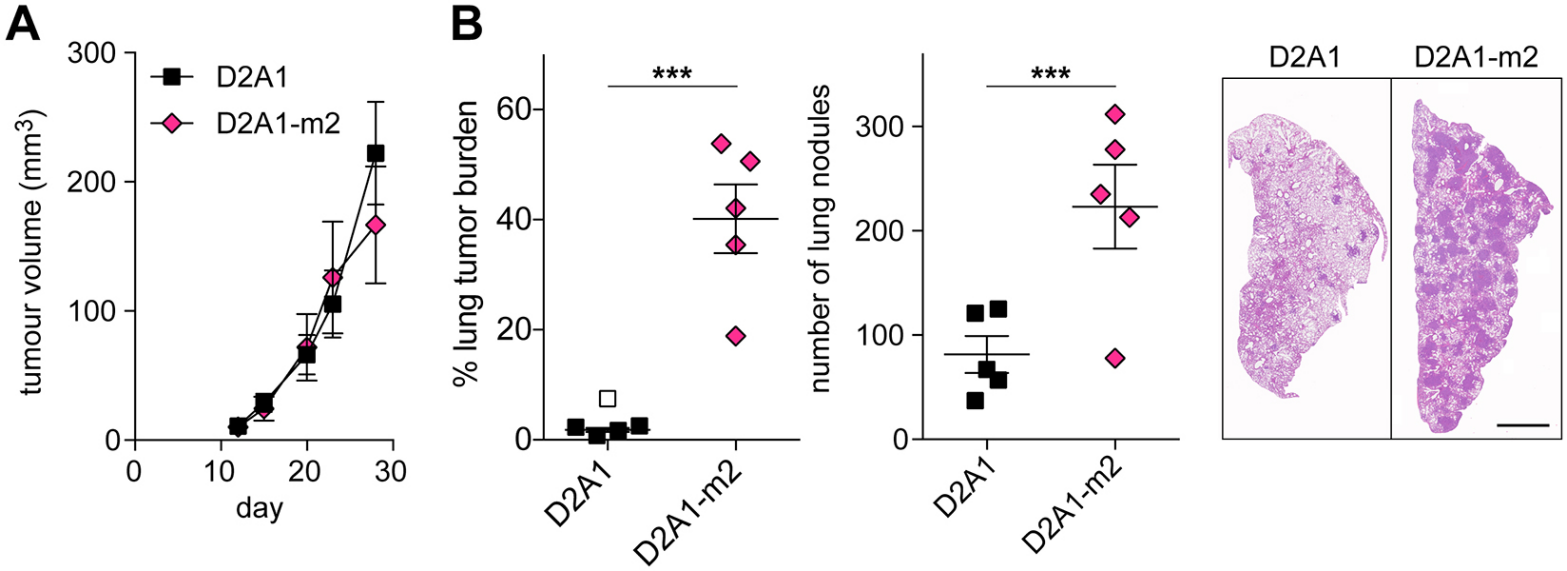
**Spontaneous metastasis in an immunocompromised setting.** 5×10^4^ D2A1 or D2A1-m2 cells were inoculated into the fourth mammary fat pad of NOD scid gamma (NSG) mice (*n*=5 mice per group). (A) Mean tumour volume ±s.e.m. (nonsignificant at all time points). All mice were culled on day 28 when the first mouse showed signs of ill health. (B) Metastatic burden in the lung assessed as % tumour area and number of metastatic nodules per lung section. Data are mean values per mouse ±s.e.m. Significant outlier is shown as a white symbol. Representative images are shown (right). Scale bar: 2 mm. ***P<0.001.

To assess whether the immune system plays a role in limiting D2A1 tumour growth and metastasis, parental D2A1 cells and the metastatic D2A1-m2 subline were inoculated orthotopically into immunocompetent BALB/c (Fig. S1) or immunocompromised NOD scid gamma (NSG) (Fig. 2) mice. In both mouse strains, there was no significant difference in primary tumour growth but, again, the D2A1-m2 cells gave rise to a significantly increased metastatic burden in the lung. Moreover, although the primary tumours did not grow at a noticeably faster rate in the NSG compared to the BALB/c mice (Fig. 2A; Fig. S1A), it is important to note that the NSG mice experiment had to be terminated at an earlier time (and when the primary tumours were of a much smaller size) owing to the extensive burden of metastatic disease in the D2A1-m2-inoculated mice (Fig. 2B). In addition, although the D2A1 parental cells showed a much reduced metastatic burden compared to the D2A1-m2 subline, the amount of D2A1 metastatic burden in the lung was greater in the immunocompromised NSG mice compared to the BALB/c mice. Although differences in tumour growth can vary between different mouse strains (Hunter, 2006), these data suggest that both the D2A1 parental cells and their metastatic derivatives are under immune control, particularly in the metastatic setting, and that evasion of immune control cannot fully explain the enhanced metastatic properties of the metastatic subline.

### Experimental metastasis assays

**Fig. 3.**
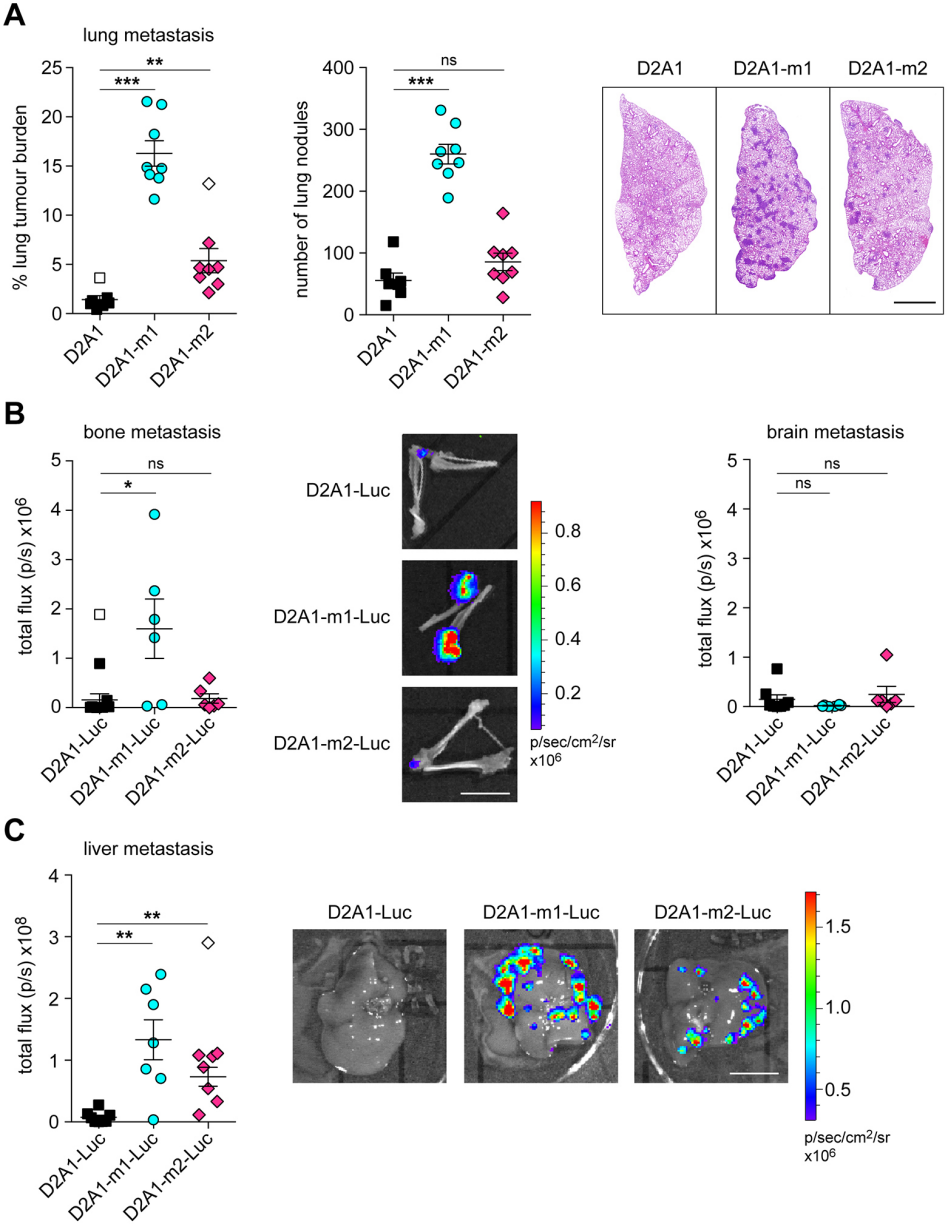
**Experimental metastasis assays.** (A) 4×10^5^ D2A1, D2A1-m1 or D2A1-m2 cells were inoculated via the tail vein into BALB/c mice (*n*=8 mice per group). 11 days later, lungs were removed at necropsy. Data show quantification of tumour burden as monitored by % tumour area and number of lung nodules per lung section. Representative lung images are shown (right). Scale bar: 2 mm. (B) 2×10^5^ D2A1-Luc, D2A1-m1-Luc or D2A1-m2-Luc cells were inoculated into the left ventricle of BALB/c mice (*n*=6 mice per group). 10 days later, bones and brains were removed at necropsy and IVIS imaged *ex vivo*. Representative *ex vivo* bone IVIS images are shown (middle). Scale bar: 1 cm. (C) 2×10^5^ D2A1-Luc, D2A1-m1-Luc or D2A1-m2-Luc cells were inoculated into the spleen of BALB/c mice (*n*=8 mice per group). 13 days later, livers were removed at necropsy and IVIS imaged *ex vivo*. Representative IVIS images are shown (right). Scale bar: 1 cm. Significant outliers are shown as white symbols. All data are mean values per mouse ±s.e.m. *P<0.05; **P<0.01; ***P<0.001; ns, nonsignificant.

Next, we assessed the ability of the two metastatic sublines to colonise secondary sites in different experimental metastasis assays. First, untagged (Fig. 3A) or luciferase-tagged (Fig. S2) tumour cells were inoculated intravenously into recipient BALB/c mice. Consistent with the spontaneous metastasis assays, injection of both sublines resulted in a greater lung tumour burden than from the parental cells. Interestingly, although the D2A1-m2 subline gave rise to a higher burden of spontaneous lung metastases compared to the D2A1-m1 subline (Fig. 1B), in this experimental metastasis assay it was the D2A1-m1 subline that gave rise to the greatest lung tumour burden (Fig. 3A).

Intravenous inoculation results in the majority of tumour cells lodging in the lungs. To investigate other sites of metastasis, we next performed intracardiac inoculation of tumour cells (Fig. 3B), which favours dissemination via the arterial system to the bones, brain and other organs (Bos et al., 2009; Khanna and Hunter, 2005). *Ex vivo* IVIS imaging revealed that, again, the D2A1-m1 subline gave rise to the greatest tumour burden in the bones. Neither the D2A1 parental cells nor the metastatic sublines showed evidence of brain colonisation.

Finally, we performed intrasplenic inoculations to assess colonisation of the liver (Khanna and Hunter, 2005) (Fig. 3C). We observed that 50% of mice inoculated with parental D2A1 cells had undetectable tumour burden in the liver, but both the D2A1-m1 and D2A1-m2 sublines gave rise to extensive disease, with only one mouse in each group remaining tumour free.

Together, these *in vivo* experiments indicate that the two D2A1 sublines have different properties, with the D2A1-m1 subline having a superior ability to grow in the lungs and the bones, but, compared to the D2A1-m2 subline, a reduced ability to disseminate from the primary tumour. Consequently, we addressed whether these two sublines displayed altered properties using a panel of *in vitro* proliferation, migration and invasion assays.

### D2A1 metastatic sublines in adherent culture

**Fig. 4.**
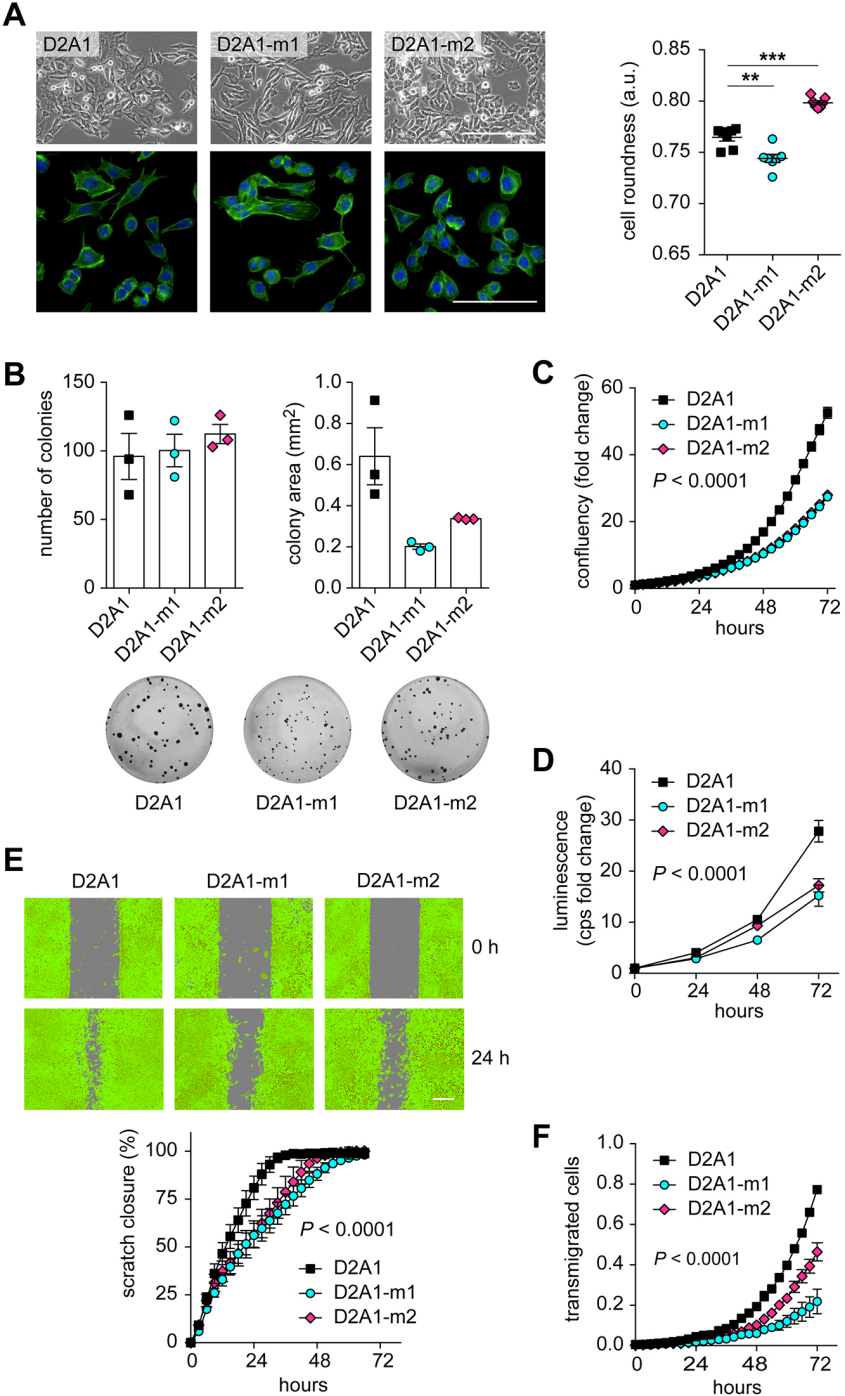
**Characterisation of the D2A1 metastatic sublines *in vitro*.** (A) Upper row: phase contrast images of D2A1, D2A1-m1 and D2A1-m2 cells. Scale bar: 200 µm. Lower row: cells cultured on coverslips were stained with Alexa Fluor 488-phalloidin and DAPI. Scale bar: 100 µm. Data are mean values (*n*=6 samples per cell line) ±s.e.m. of cell roundness in arbitrary units (see Materials and Methods). Equivalent results were obtained on two separate occasions. **P<0.01; ***P<0.001. (B) Colony formation assay. Data are mean number of colonies and average colony area ±s.e.m., with *n*=3 wells per cell line. Representative images of Crystal Violet-stained wells are shown below. Equivalent results were obtained on three separate occasions. (C) Cell proliferation monitored using the IncuCyte Live-Cell Analysis System. Data are mean values ±s.d., with *n*=6 wells per cell line. Equivalent results were obtained on two separate occasions. (D) Cell viability measured by CellTiter-Glo at the indicated time points. Data are mean values ±s.d. (fold change in luminescence in counts per second), with *n*=6 wells per cell line per time point. Equivalent results were obtained on two separate occasions. (E) Wound healing assay monitored using the IncuCyte Live-Cell Analysis System. Data represent % wound closure ±s.d., with *n*=8 wells per cell line. Representative false-coloured images of cells at 0 h and 24 h are shown. Scale bar: 300 µm. (F) Transwell chemotaxis assay. Data are mean values ±s.e.m. (*n*=3) for cells transmigrated to the lower side of the filter relative to the initial plated cells. In C-F, statistical differences were determined using two-way ANOVA and Bonferroni post-hoc testing, with time and cell lines as independent variables. In all cases, both D2A1-m1 and D2A1-m2 are significantly different to the parental D2A1 cells (*P*<0.0001).

When growing as adherent cultures on tissue culture plastic the D2A1 metastatic sublines have distinct morphologies, with D2A1-m1 cells having a more elongated shape, and D2A1-m2 cells being more rounded, compared to the parental cells (Fig. 4A; Fig. S3). These different morphologies did not impact on the plating efficiency as exemplified by all three cell lines giving rise to the same number of colonies in a colony formation assay (Fig. 4B). By contrast, in this assay, it was notable that the two metastatic sublines gave rise to smaller colonies, indicating a lower proliferation rate *in vitro*. Consistent with this, both sublines displayed a lower proliferation rate as continuously monitored in the IncuCyte live-cell analysis (Fig. 4C) and reduced cell number over time as monitored in the CellTiter-Glo assay (Fig. 4D). Together with the observation that there was no consistent difference in primary tumour growth between the parental line and the sublines, these data indicate that the increased metastatic ability of the sublines is not caused by acquisition of a more proliferative phenotype.

Next, we tested whether the sublines show a difference in their migratory abilities, a characteristic linked to an increased metastatic potential. To our surprise, both sublines displayed a reduced migratory and chemotactic capacity *in vitro*, as monitored in the IncuCyte scratch wound assay (Fig. 4E) and a Transwell chemotactic assay (Fig. 4F). These data highlight how morphology and cell behaviour in 2D adherent assays cannot be used as a predictor of metastatic behaviour *in vivo*. Consequently, we performed assays under non-adherent conditions.

### D2A1 metastatic sublines in non-adherent culture

**Fig. 5.**
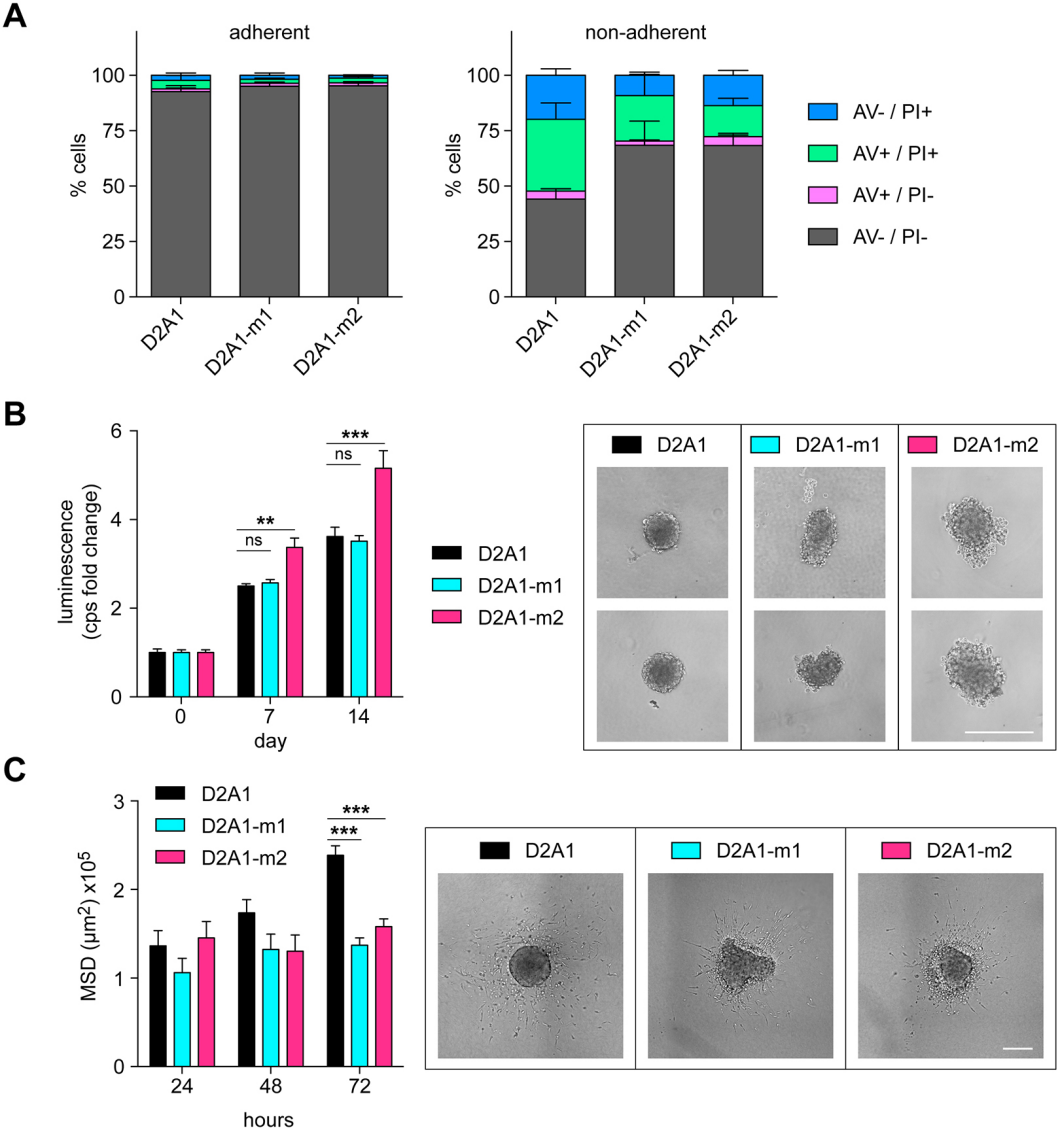
**Characterisation of the D2A1 metastatic sublines in non-adherent culture.** (A) D2A1, D2A1-m1 and D2A1-m2 cells were plated onto adherent tissue culture or non-adherent six-well plates and apoptosis was monitored 24 h later by annexin V/PI staining. Data show the proportions of live (AV−/PI−), early apoptotic (AV+/PI−), late apoptotic (AV+/PI+) and necrotic (AV−/PI+) cells, as a mean of three experiments ±s.e.m., with *n*=3 per cell line per experiment. D2A1 cells had significantly fewer viable cells compared to D2A1-m1 (*P*=0.0114) and D2A1-m2 (*P*=0.0119) cells. (B) Spheroid assay. Cells were plated in U-bottom low-adherence plates (*n*=6 per cell line) and cultured for 14 days. At stated time points, cell viability in the tumour spheroids was monitored by CellTiter-Glo. Data are mean values (fold change in luminescence in counts per second) ±s.e.m. Examples of tumour spheroids at day 14 are shown (right). Scale bar: 400 µm. Equivalent results were obtained on two separate occasions. (C) Invasion into a 3D collagen matrix. Spheroids were embedded into collagen and single cell invasion was monitored 24, 48 and 72 h later by measuring the mean square displacement (MSD). Data are mean values ±s.e.m., with *n*=3-6 spheroids per time point. Representative images at 48 h are shown (right). Scale bar: 400 µm. In all panels, statistical differences were determined using two-way ANOVA and Bonferroni post hoc testing. Equivalent results were obtained on two separate occasions. **P<0.01; ***P<0.001; ns, nonsignificant.

As an example of how non-adherent assays can reveal properties of cells that are not evident in adherent assays, D2A1 parental cells and the D2A1 metastatic sublines were seeded into tissue culture or low-adherence plates and monitored for apoptosis after 24 h (Fig. 5A). In adherent culture, essentially all cells were viable as monitored by the lack of annexin V (AV) staining and propidium iodide (PI) uptake. After 24 h in non-adherent culture, >50% of the D2A1 parental cells were classed as early apoptotic (AV+/PI−), late apoptotic (AV +/PI+) or necrotic (AV−/PI+), while both the D2A1-m1 and D2A1-m2 sublines showed a significantly lower level of anoikis under these conditions. To explore this further, we next plated cells into low-adherence U-bottom plates (Fig. 5B). The parental cells readily assembled into dense tumour spheroids with well-delineated margins, and expanded in size over time. Of the two metastatic sublines, the D2A1-m1 subline again assembled into tumour spheroids but, consistently, they were less regular in shape. However, there was no difference in D2A1-m1 spheroid growth compared to the parental cells. By contrast, the D2A1-m2 subline formed less well-aggregated spheroids with loosely attached cells associated with the main core. Moreover, as monitored by CellTiter-Glo, these disorganised spheroids showed a significant increase in cell number over time compared to the parental D2A1 and D2A1-m1 spheroids.

Given the less regular spheroid shape formed by the D2A1 sublines, we anticipated that the more loosely attached tumour cells might be more invasive. To address this, tumour spheroids were transferred after 3 days into a collagen matrix. At the indicated time points, collagen plugs were fixed, stained with DAPI, and cell invasion into the 3D collagen matrix was quantified (Fig. 5C). Surprisingly, the parental D2A1 cells were significantly more invasive than either of the metastatic sublines. Despite the limitation of using *in vitro* assays to model *in vivo* metastasis, these data point to the metastatic sublines having an enhanced metastatic capacity owing to their ability to resist stress-induced anoikis.

### D2A1-m2 cells promote stromal cell activation

**Fig. 6.**
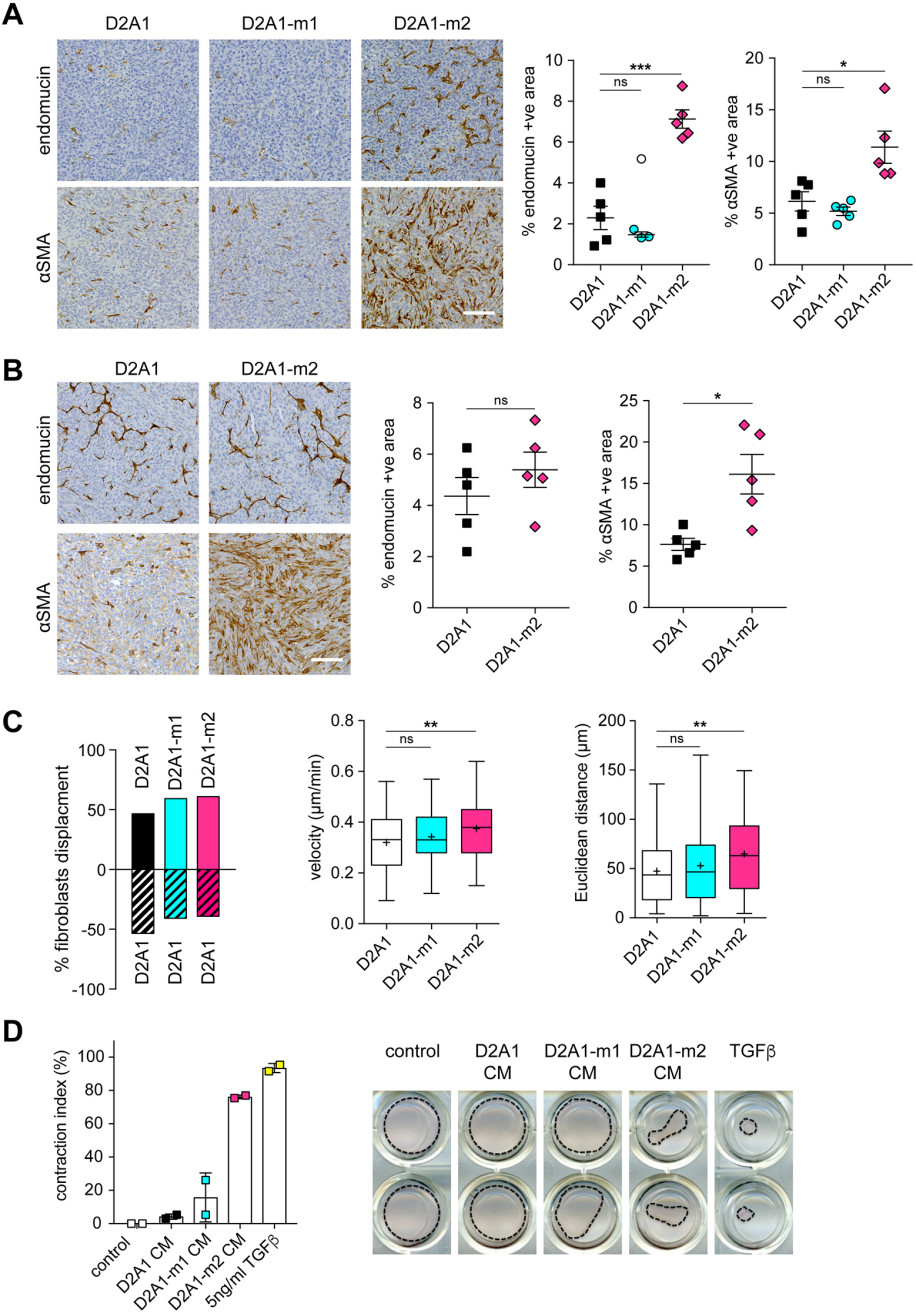
**Stromal cell recruitment in primary tumours.** (A,B) Primary tumours from the spontaneous metastasis experiments shown in Fig. 1B and Fig. 2 were sectioned and stained for αSMA or endomucin. (A) BALB/c mice from Fig. 1B; (B) NSG mice from Fig. 2. Representative images are shown (left). Scale bars: 100 µm. Quantification of staining from at least four tumours per group ±s.e.m. (*n*>6 fields of view per tumour) (right). Significant outlier is shown as a white symbol. (C) Competitive fibroblast attraction assay (see Fig. S4A for experimental set up). Migration of individual 3T3 cells towards D2A1 versus D2A1, D2A1 versus D2A1-m1, or D2A1 versus D2A1-m2, cells was monitored over 8 h (*n*>60 cells tracked/sample). Left panel: percentage net displacement of fibroblasts (see Fig. S4B for individual cell tracks and quantification). Middle and right panels: velocity (µm/min) and Euclidean distance (µm) of fibroblast migration. In box and whisker graphs, boxes extend from the 25th to 75th percentiles, middle lines are plotted at the median, whiskers are minimum and maximum, and+indicates the mean of data. (D) Fibroblast contraction assay. Data are % contraction of the fibroblast containing Matrigel/collagen gel after 14 days treatment with DMEM, TGFβ or conditioned medium from D2A1, D2A1-m1 and D2A1-m2 cells, with *n*=2 wells/condition. Representative images of the gels are shown (right). *P<0.05; **P<0.01; ***P<0.001; ns, nonsignificant.

To investigate further how these differing behaviours in non-adherent culture might impact on tumour growth *in vivo*, primary tumours in BALB/c mice (Fig. 1B; Fig. S1A) and NSG mice (Fig. 1C) were sectioned and stained for αSMA (Acta2), a marker of activated fibroblasts, and endomucin, a marker of endothelial cells. The elevated levels of αSMA-positive fibroblasts and blood vessels in the D2A1-m2 primary tumours in both the BALB/c immunocompetent (Fig. 6A; Fig. S1D,E) and NSG immunocompromised (Fig. 6B) mice suggest that this subline has an enhanced ability to promote stromal cell recruitment activation and this could, in part, provide a mechanistic explanation for the enhanced spontaneous dissemination of tumour cells to secondary sites.

Although stromal cell activation and recruitment observed in *in vivo* tumours cannot be fully recapitulated *in vitro*, we examined whether the metastatic sublines had an increased ability to promote directional migration of fibroblasts using the ‘ibidi’ µ-slide chemotaxis system. To do this, 3T3 mouse fibroblasts were plated into the central viewing chamber that is connected to two larger reservoirs on either side (Fig. S4A). By plating different cell populations into the two reservoirs, conditioned medium gradients are set up and cell migration can be monitored by time-lapse microscopy. As expected, if D2A1 cells are plated into both reservoirs, there is no bias in the directional migration of the fibroblasts (Fig. 6C, left panel). However, if D2A1 cells are plated into one reservoir and either D2A1-m1 or D2A1-m2 cells are plated into the other, the fibroblasts show preferential displacement towards the metastatic sublines over the parental cells (see Fig. S4B for individual fibroblast migration tracks). However, albeit with relatively small effects, only the D2A1-m2 subline induced a significant difference in fibroblast migration speed (velocity) and Euclidean distance migrated (Fig. 6C, middle and right panels) and a significant fibroblast chemotaxis (Rayleigh test; Fig. S4C). Finally, to assess the capacity of the metastatic sublines to promote fibroblast activation, fibroblasts were embedded into a Matrigel/collagen gel and treated with conditioned medium from D2A1, D2A1-m1 or D2A1-m2 cells or with TGFβ as a positive control (Fig. 6D). Only D2A1-m2 conditioned medium and TGFβ treatment resulted in a significant induction of fibroblast contractility. Consistent with the *in vivo* observations (Fig. 6A), these data indicate that the D2A1-m2 subline has an enhanced ability to both recruit and activate stromal fibroblasts.

### Gene expression profiling and analysis of human datasets

**Fig. 7.**
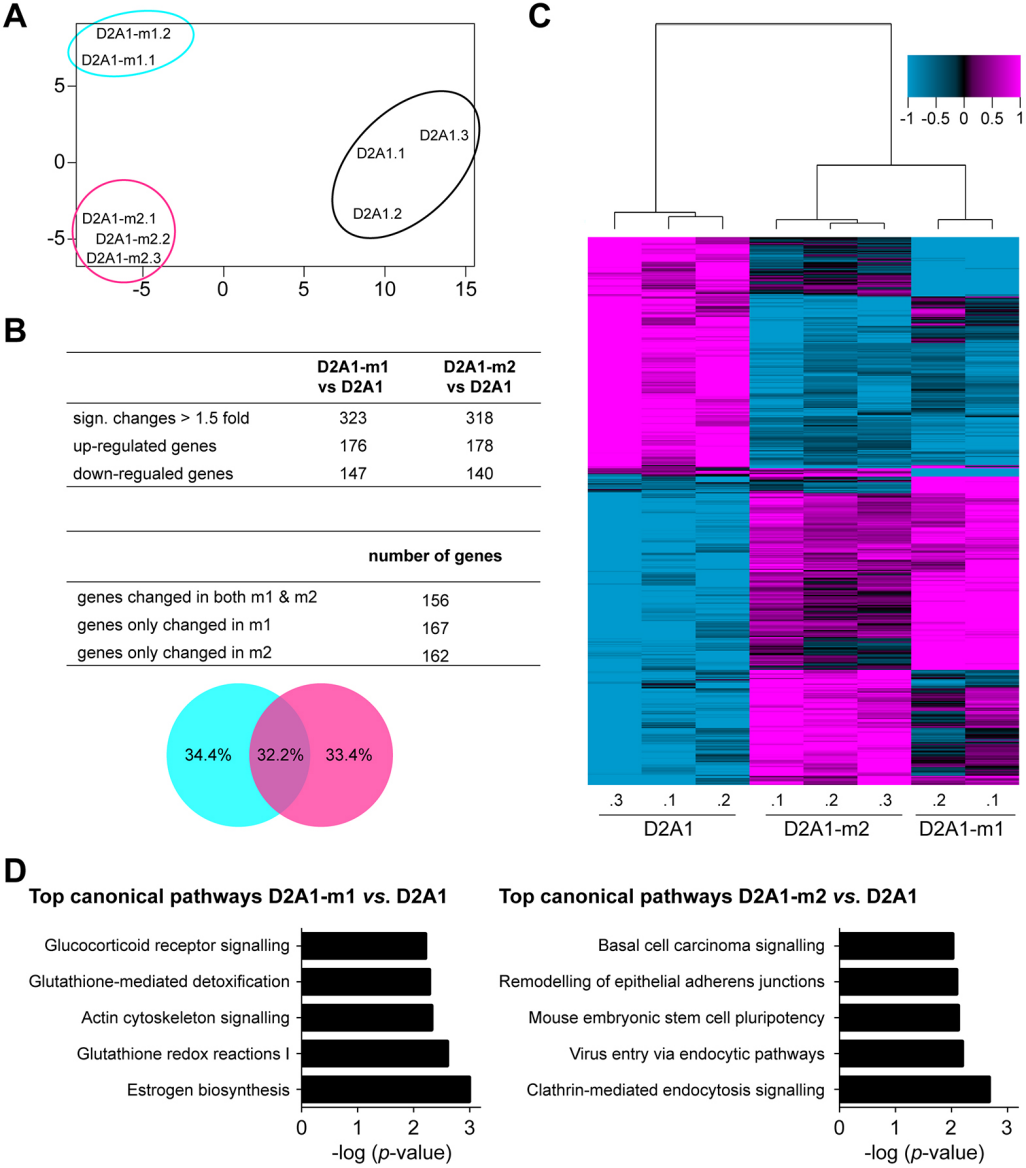
**Gene expression profiling.** RNA was isolated from independent biological replicates (D2A1.1, D2A1.2 and D2A1.3; D2A1-m1.1 and D2A1-m1.2; D2A1-m2.1, D2A1-m2.2 and D2A1-m2.3) and subject to gene expression profiling. (A) Principal component analysis estimating the relations of D2A1, D2A1-m1 and D2A1-m2 cells based on the genes with coefficient of variance >0.1 across the eight samples. (B) The number of differentially expressed genes (*P*<0.001, ≥1.5-fold change), for D2A1-m1 versus D2A1, and D2A1-m2 versus D2A1, is shown in the top panel. The number of unique and shared differentially expressed genes between D2A1-m1 and D2A1-m2 sublines is shown in the middle panel. The bottom panel is a Venn diagram depicting percentages. (C) Dendrogram shows correlation-centred hierarchical clustering based on average linkage. Tumour cell expression data of 481 genes that were significantly differentially expressed between D2A1 and D2A1-m1, or D2A1 and D2A1-m2, cells with ≥1.5-fold change (*P*<0.001) are shown. (D) The top canonical pathways changed between D2A1-m1 and D2A1, as well as D2A1-m2 and D2A1, identified using IPA (*P*<0.05) based on the differentially expressed genes (*P*<0.001, ≥1.5-fold change).

Finally, we subjected the parental D2A1 cells and metastatic sublines to gene expression profiling. Principal component analysis revealed that each cell line was qualitatively unique, with the metastatic sublines being distinct, but more closely related to each other than to the parental cells (Fig. 7A). Overall, there were 890 genes differentially expressed between the D2A1-m1 cells and parental D2A1 cells, of which 323 genes were with a fold change of ≥1.5. In the case of the D2A1-m2 cells, 1339 genes were differentially expressed, of which 318 had a fold change ≥1.5 (Fig. 7B). A comparison of the differentially expressed genes showed that ∼50% were unique to each subline and 50% overlapped as shown in the Venn diagram (Fig. 7B, bottom panel). Unsupervised 2D hierarchical clustering of the D2A1, D2A1-m1 and D2A1-m2 cells (based on genes with a fold change ≥1.5, using their log2 gene expression) confirmed the close relationship of the two sublines but also distinct gene expression patterns (Fig. 7C). Top differentially expressed genes for D2A1-m1 versus D2A1, and D2A1-m2 versus D2A1, are shown in Tables S1 and S2, respectively. Top genes that were differentially changed in both sublines (Fig. 7B) and their average fold change are shown in Table S3. Similarly, Ingenuity Pathway Analysis (IPA) demonstrated distinct top canonical pathways, while the upstream regulators and molecular and cellular functions were highly overlapping (Fig. 7D; Tables S4 and S5). Even though in both D2A1-m1 and D2A1-m2 cells, TGFβ1 is identified as an upstream regulator, only in D2A1-m2 subline is the predictive *z*-score >2-fold, indicating a stronger activation of this pathway in D2A1-m2 cells than in the parental and D2A1-m1 cells, and potentially accounting for the observed ability of the D2A1-m2 subline to promote stromal activation.

## DISCUSSION

Studying the complex mechanisms driving breast cancer metastasis and therapeutic response of advanced disease has been hampered by the lack of robust *in vivo* models, and in particular spontaneously metastatic syngeneic models with which the interaction with an intact immune system can be studied. To date, there has been heavy reliance on the BALB/c-derived 4T1 cell line (Aslakson and Miller, 1992; Fantozzi and Christofori, 2006) as 4T1 cells, and selected 4T1 sublines, readily form primary orthotopic tumours that can spontaneously metastasise to the lungs and other secondary sites (Eckhardt et al., 2005; Fantozzi and Christofori, 2006; Yang et al., 2004). More recently, Johnstone and colleagues have described the generation of a spontaneously metastatic E0771.LMB subline from the C57BL/6-derived E0771 mouse medullary mammary adenocarcinoma E0771 cell line (Johnstone et al., 2015) that provides a valuable model in a different mouse strain (Chen et al., 2017). The molecular and phenotypic characterisation of 12 mouse mammary carcinoma cell lines (Yang et al., 2017) provides valuable information for the research community, but, as the authors note, the efficiency of metastatic spread with these lines lacks reproducibility; for example, only 45% of the mice inoculated with parental D2A1 cells had detectable metastatic lesions in the lungs. Alternatives to syngeneic mouse cell lines are tumour-prone genetically modified mice (Fantozzi and Christofori, 2006), such as those expressing a polyoma middle T (PyMT) (Guy et al., 1992a) or ErbB2/neu (Guy et al., 1992b) transgene in the mammary gland. Genetically modified models have provided powerful insights into tumour initiation and progression, but they have limitations when studying the development of distant metastases, owing to the variable latency and often late presentation of secondary disease, and the inability to rapidly perform additional genetic manipulation. In addition to the mouse syngeneic models, there are many human breast cancer cell lines that can be grown as xenografts in immunocompromised mice, and, more recently, an increasing number of patient-derived xenografts in which primary tumour material is transplanted directly into immunocompromised mice and then serially passaged. Such models have been extremely valuable for studying the heterogeneity of human disease and for monitoring response to tumour-targeting agents (Bruna et al., 2016; Byrne et al., 2017; Holen et al., 2017; Neve et al., 2006). However, few of these models reproducibly spontaneous metastasise. Further, the lack of a species-matched stroma, and the lack of an intact immune system, limit the interrogation of breast cancer biology and/or the assessment of stromal targeting agents. Consequently, there remains an urgent need to develop a wider variety of syngeneic models with which the different stages of the metastatic cascade can be investigated.

In this study, we describe the generation and characterisation of two sublines of the poorly metastatic mouse mammary tumour cell line, D2A1. These sublines, D2A1-m1 and D2A1-m2, reproducibly give rise to spontaneous metastases from the primary tumour to the lungs, and in experimental metastasis assays readily colonise the lung, liver and, in case of the D2A1-m1 subline, bone. However, there are notable differences in the behaviour of these two sublines *in vivo*. In particular, the D2A1-m1 subline, compared to the D2A1-m2 subline, is less efficient at disseminating from the primary tumour in a spontaneous metastasis assay, but more efficient at colonising the lungs, bone and liver when inoculated via the tail vein, the left ventricle of the heart or the spleen, respectively. This would suggest that the D2A1-m1 subline has acquired properties that better enable it to extravasate from the circulation and/or efficiently proliferate at these secondary sites and that, conversely, the D2A1-m2 subline has acquired properties that better enable it to disseminate from the primary tumours.

To address the potential mechanism underlying these distinct phenotypes, first we extensively characterised these cells lines in *in vitro* assays. In adherent cultures, the two sublines have distinct morphologies, with the D2A1-m1 cells having a more elongated, and the D2A1-m2 cells have a more rounded, shape. However, these morphological differences did not relate to their behaviour, with both sublines, surprisingly, showing a lower rate of proliferation and migration compared to the parental D2A1 cells. More revealing was their behaviour in non-adherent conditions. When normal epithelial cells are detached from a matrix, a programme of caspase-mediated apoptosis, known as anoikis, is activated (Frisch and Screaton, 2001; Paoli et al., 2013). A hallmark of cancer is ‘resisting cell death’ (Hanahan and Weinberg, 2011), and D2A1 parental cells show evidence of such resistance as evidenced by their ability to form and proliferate as free-floating tumour spheroids when plated into U-bottom low-attachment plates. However, when plated under conditions that do not promote cell-cell contact between the detached cells (low-adherent flat-bottomed plates), within 24 h, >50% of the D2A1 cells show markers of apoptotic cell death, compared to <10% of the cells plated onto adherent flat-bottomed plates. In the same assays, both metastatic sublines show increased resistance to anoikis, and in spheroid culture, the D2A1-m2 cells show enhanced proliferative ability. Consequently, we conclude that the increased metastatic potential of the two sublines is caused, at least in part, by their ability to survive in a more hostile environment. However, these *in vitro* studies were relatively uninformative as to why the D2A1-m1 subline shows a more aggressive behaviour in experimental metastasis assays. Some clues are provided by gene expression profiling, in which examination of genes differentially expressed between the D2A1-m1 subline and the parental cells revealed the upregulation of canonical pathways involved in glutathione regulation and the actin cytoskeleton, features that could provide an advantage for the cells in extravasating from the circulation and/or surviving in the foreign metastatic environments. Future studies will be required to fully address this hypothesis, and it will certainly be of interest to compare the transcriptional profile of the D2A1 sublines freshly isolated from tumours, as this might well be more informative that profiling the sublines grown in culture.

More informative was the analysis of the D2A1-m2 subline. In addition to showing an increased resistance to anoikis, the D2A1-m2 primary tumours, in both immunocompetent BALB/c mice and immunocompromised NSG mice, showed striking infiltration of αSMA-positive stromal fibroblasts and pericytes. At least in part, this phenotype could be recapitulated *in vitro*, showing that when presented with an option for directional migration, cultured fibroblasts preferentially migrate, at an increased velocity, towards the D2A1-m2 subline and D2A1-m2 conditioned medium, but not conditioned medium from D2A1 or D2A1-m1 cells, to promote fibroblast activation, as monitored in a gel contraction assay. A number of studies have demonstrated that an activated stroma, in particular the presence of activated pericytes on the blood vessels, is required for efficient intravasation of tumour cells from the primary tumour into the circulation (Harney et al., 2015; Viski et al., 2016; Xian et al., 2006; Yang et al., 2016), and activated cancer-associated fibroblasts can prime tumour cells for metastatic growth (Zhang et al., 2013). The data presented here suggest that the increased efficiency of the D2A1-m2 subline to spontaneously metastasise might, at least in part, result from the ability to recruit and activate stromal cells, particularly stromal fibroblasts and pericytes.

In summary, the data presented here describe the derivation and characterisation of two new syngeneic metastatic mouse mammary carcinoma cell lines that have both overlapping and distinct behaviours when introduced into mice. Surprisingly, the ability of these cells to give rise to both spontaneous and experimental metastases *in vivo* is not reflected by a more aggressive phenotype in *in vitro* proliferation, migration and invasion assays. However, some clues as to the mechanisms driving the metastatic phenotype have been revealed by assessing the cell lines in non-adherent culture and by examination of the tumour stroma.

## MATERIALS AND METHODS

### *In vivo* studies

All *in vivo* studies were performed under UK Home Office Project Licences 70/7413 and P6AB1448A granted under the Animals (Scientific Procedures) Act 1986. All studies were performed at The Institute of Cancer Research (Establishment Licence, X702B0E74 70/2902). Ethical permission was granted by the Institute of Cancer Research ‘Animal Welfare and Ethical Review Body’ (AWERB). Female BALB/c and NSG mice (Charles River) between 6 and 12 weeks of age were housed in individually ventilated cages, monitored on a daily basis for signs of ill health, and had food and water *ad libitum*. In all cases, experiments were terminated if the primary tumour reached a maximum allowable diameter of 15 mm or if a mouse showed signs of ill health.

The two metastatic sublines were generated independently (Fig. 1A). In each case, 5×10^4^ D2A1 cells were injected into the fourth mammary fat pad of a single BALB/c mouse under general anaesthesia. When the primary tumour reached 12-14 mm in diameter, lungs were dissected postmortem, mechanically dissociated and placed into culture in Dulbecco's modified Eagle's medium (DMEM; Gibco by ThermoFisher) plus 10% foetal bovine serum (FBS; Gibco by ThermoFisher) and 1% penicillin/streptomycin. The medium was changed after 24 h and then twice a week. After 2 weeks, when tumour cell colonies were visible, cells were replated and expanded. Once expanded, 5×10^4^ cells were injected via the tail vein into a single BALB/c mouse. After 11-13 days, lungs were processed as before. The intravenous inoculation was repeated a further two times, each time into a single mouse. This procedure resulted in the two independently selected D2A1 sublines, D2A1-m1 and D2A1-m2.

For spontaneous metastasis assays, 5×10^4^ cells were injected into the fourth mammary fat pad of BALB/c or NSG mice under general anaesthesia. Tumour growth was monitored using callipers and tumour volume calculated as 0.5236×[(width+length)/2]^3^ (Janik et al., 1975). Primary tumours and lung tissue were formalin-fixed and paraffin-embedded (FFPE) prior to sectioning. FFPE sections of primary tumours were stained with αSMA (Sigma-Aldrich, clone 1A4, 1:1000 dilution) or endomucin (Santa Cruz Biotechnology, SC-65495; 1:2000 dilution). Detection was achieved with the VectaStain ABS system and sections were scanned on a NanoZoomer Digital Pathology scanner (Hamamatsu). Horseradish peroxidase staining was analysed in ImageJ from ≥6 fields of view per tumour, avoiding areas of necrosis. To quantify lung tumour burden, sections were taken midway through the lung and stained with Haematoxylin and Eosin (H&E). Sections were scanned and analysed using the NanoZoomer Digital Pathology scanner, file names were blinded, and the number of macroscopic tumour nodules (defined as having a minimum area of 1000 µm^2^/nodule) were counted manually or quantified as percentage tumour burden. The percentage lung tumour burden was defined as (total tumour area)/(lung area)×100 from a coronal H&E section of the lung. Where indicated, primary tumours were surgically resected under general anaesthesia.

For intravenous inoculation, 4×10^5^ cells were injected into the tail vein of BALB/c mice. After 11 days, mice were sacrificed. Lungs were processed as described in the spontaneous metastasis assay. For intracardiac inoculation, 2×10^5^ luciferase expressing cells were injected into the left ventricle of BALB/c mice under general anaesthesia. Tumour burden was analysed by *ex vivo* IVIS imaging. Mice were injected intraperitoneally with 150 mg/kg D-luciferin (Caliper Life Sciences) in 100 µl. After 5 min, dissected hind limb long bones and brains were imaged using an IVIS imaging chamber (IVIS Illumina II). Luminescence measurements (photons/second/cm^2^) were acquired over 1 min and analysed using Living Image software (PerkinElmer) by placing a constant size region of interest over the tissues. For intrasplenic inoculation, 2×10^5^ luciferase expressing cells were inoculated into the spleen parenchyma of BALB/c mice under general anaesthesia. After 10 min, a splenectomy was performed to avoid growth of splenic tumours. Tumour burden in the liver was assessed by *ex vivo* IVIS imaging as described above.

### Cell lines

D2A1 cells were from Chambers laboratory stocks, from stocks originally obtained from Dr Fred Miller (Mahoney et al., 1985; Miller et al., 1989). NIH-3T3 cells were from Isacke laboratory stocks. Cells were maintained in DMEM plus 10% FBS and 1% penicillin/streptomycin. Cells were luciferase transduced with lentiviral expression particles containing a firefly luciferase gene and a blasticidin-resistance gene (Amsbio, LVP326). All cells were routinely subject to mycoplasma testing.

### *In vitro* studies

For cell shape analysis, 1×10^3^ cells/well were seeded into a ViewPlate-96 Black with optically clear bottom (PerkinElmer). After 24 h, cells were fixed in 4% paraformaldehyde (PFA) and permeabilised with 0.5% Triton X-100, prior to staining with DAPI (Molecular Probes, D1306, 1:10,000) and Alexa Fluor 488-labelled phalloidin (Molecular Probes, A12379, 1:500). Automated image acquisition was performed on an Operetta high content imaging system (Perkin Elmer). Cell shapes were analysed using basic algorithms in the Harmony high content analysis software package (PerkinElmer). Cells were initially defined using the DAPI channel to identify the nucleus, and the cytoplasm was segmented using the Alexa Fluor 488 channel. After this, the Harmony software allows the extraction of cell shape parameters, including ‘cell roundness’. On average, 1110 cells were analysed per well (*n*=6 wells/cell line).

For cell proliferation/viability assays, 1×10^3^ cells/well were seeded into 96-well plates. Cell viability was quantified either by CellTiter-Glo (Promega) at the indicated time points or by time-lapse imaging and quantification of cell confluence using the Live-Cell Analysis System IncuCyte (EssenBioscience).

For colony formation assay, 50 cells/well were seeded into a six-well plate. Tumour cell colonies were stained 7 days later with Crystal Violet. Plates were scanned using a GelCount (Oxford Optronix), and image analysis was performed using GelCount software and ImageJ.

For scratch wound migration assays, cells were seeded at high density to form a confluent layer in a 96-well Image Lockplate (EssenBioscience). Scratches were created using a WoundMaker tool (EssenBioscience). Plates were imaged for 72 h and analysed using the Live-Cell Analysis System IncuCyte (EssenBiosience).

For the Transwell migration assay, 1×10^3^ cells/well were seeded into IncuCyte ClearView 96-well chemotaxis plates in DMEM supplemented with 3% FBS. The bottom well contained DMEM supplemented with 10% FBS. Chemotactic migration was monitored and analysed using the IncuCyte Chemotaxis System (EssenBiosience).

For competitive fibroblast attraction assays, the µ-Slide Chemotaxis system (ibidi) was used. In brief, 1.2×10^4^ 3T3 fibroblasts were seeded into the central observation chamber. After 3 h, 3×10^4^ D2A1 cells were seeded into the left reservoir and either D2A1, D2A1-m1 or D2A1-m2 cells were seeded into the right reservoir. Migration of 3T3 fibroblasts was imaged over 8 h (20 min intervals) using Slidebook 6 (3i) and a Nikon Eclipse TE2000-5 widefield microscope equipped with a plan fluor 10×/0.3 NA PH1 WD 16 mm objective (Nikon) and a temperature- and CO_2_-controlled chamber. Cell migration was analysed using the ImageJ Manual Tracking module and Chemotaxis and Migration Tool (ibidi) plugin.

To collect conditioned medium, cells were seeded in DMEM supplemented with 10% FBS and cultured until 70-80% confluency. The medium was then changed to DMEM supplemented with 2% FBS. Conditioned medium was collected after 24 h and filtered prior to use. For fibroblast contraction assays, 7×10^4^ 3T3 fibroblasts were embedded in 100 µl of a Matrigel (final concentration 2 mg/ml, Corning) and rat tail Collagen I (final concentration 4 mg/ml, Corning) mixture and seeded onto a glass-bottomed dish (P24G-1.0-13-F, MatTek Corporation). After the gel was set at 37°C, conditioned medium or DMEM supplemented with 2% FBS with or without recombinant TGFβ1 (R&D Systems) (5 ng/ml) was added. After 14 days, plates were scanned and the contracted gel area was measured using ImageJ.

For apoptosis assays, 5×10^4^ cells/well were plated into either tissue culture-treated or low-adherence six-well plates. Then, 24 h after seeding, cells were stained with the Annexin V-APC/PI Apoptosis Detection Kit (eBioscience) and analysed using a BD Biosciences LSRII flow cytometer with FACSDIVA and FlowJo software.

For spheroid growth assays, 1×10^2^ cells were seeded into ultra-low-adherence 96-well round-bottomed plates (Corning). Spheroid growth was monitored using the Celigo Image Cytometer (Nexcelom Bioscience). Cell viability was monitored with CellTiter-Glo at the indicated time points. To ensure proper lysis of the spheroids, the incubation time with the CellTiter-Glo reagent was extended from 10 to 30 min before recording luminescence.

For invasion assays, 2×10^4^ cells were seeded into ultra-low-adherence 96-well round-bottomed plates. After 3 days, spheroids were transferred into fresh round-bottomed plates containing 2 mg/ml collagen (rat tail collagen, Corning). After 24, 48 and 72 h, collagen plugs were fixed in 4% PFA, permeabilised with 0.5% Triton X-100 and stained with DAPI. Confocal *z*-stacks were acquired using the ImageXpress Micro Confocal High-Content Analysis System (Molecular Devices) equipped with a 60 µm pinhole spinning-disk, and a plan apo λ 10×/0.45 NA objective (Nikon). *Z*-stacks were maximally projected and DAPI images were quantified using a custom-written MATLAB script. In brief, spheroids were binarised using an intensity threshold, while individual cells invading into the collagen, away from the spheroid, were marked using the watershed transform, which allows separation of contacting cells. The centroid coordinates of the spheroid and individual cells were extracted and the distance *d* between each cell and the spheroid was calculated using Euclidean trigonometry. The mean square displacement (MSD), a global measure of invasion, was obtained using the following equation:
(1)
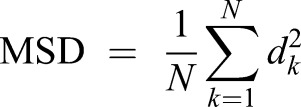
where *d_k_* is the distance (in µm) between the *k* cell and the spheroid and *N* is the total number of cells.

### Gene expression profiling and analysis of clinical datasets

RNA from D2A1 and D2A1-m2 cultured cells (*n*=3 independent biological repicates) and D2A1-m1 cultured cells (*n*=2 independent biological replicates) was extracted using an RNeasy Mini kit (Qiagen). Microarray experiments were performed in two independent batches at Cambridge Genomic Services, University of Cambridge. In brief, RNA was assessed for concentration and quality using a SpectroStar (BMG Labtech) and a Bioanalyser (Agilent Technologies). The eight RNA samples were amplified, labelled and hybridised on a MouseWG-6 v2.0 Expression BeadChip array (Illumina) following the manufacturer's instructions. Raw expression data were extracted in R using lumi package (http://www.bioconductor.org). Data were filtered to remove any non-expressed probes (detection *P*>0.01) across all samples, transformed using variance-stabilising transformation, normalised using the robust spline normalisation method, and then batch corrected using the function (ComBat) in the R package (sva). Sample relations were estimated using principal component analysis based on 7970 genes with coefficient of variance (standard deviation/mean >0.1). Two-sample *t*-tests were used to identify differentially expressed genes between (a) D2A1 and D2A1-m1, (b) D2A1 and D2A1-m2, and (c) D2A1-m1 and D2A1-m2, using BRB-Array Tools (https://brb.nci.nih.gov/BRB-ArrayTools/), with a threshold of parametric *P*<0.001. Differentially expressed genes with a fold change ≥1.5 were subjected to IPA to identify altered pathways, with *P*<0.05 considered significant. When multiple probes were mapped to the same gene, the most variable probe measured by interquartile range (IQR) across the samples was selected to represent the gene. Gene expression data from this study are deposited in NCBI Gene Expression Omnibus (GSE101579).

### Statistics

Statistics were performed using GraphPad Prism 6. Unless otherwise stated, all numerical data are expressed as mean±s.e.m. Significant outliers were identified using the Grubb's test (α=0.05, GraphPad Prism) and are shown as white symbols in the graphs. If not indicated otherwise, all comparisons between two groups were made using two-tailed, unpaired Student's *t*-test. If there was a significant difference in the variance of samples, a Welch's correction was applied. Where multiple groups over time were compared, a two-way ANOVA followed by Bonferroni post hoc testing was performed. *P<0.05; **P<0.01; ***P<0.001.

## Supplementary Material

Supplementary information
