# Supplementary material for: Generation and characterisation of two D2A1 mammary cancer sublines to model spontaneous and experimental metastasis in a syngeneic BALB/c host
Source: Dis Model Mech. 2018 Jan 1;11(1):dmm031740. doi: 10.1242/dmm.031740 (PMC5818081; doi:10.1242/dmm.031740)
Supplement: Supplementary information [file dmm-11-031740-s1.pdf]

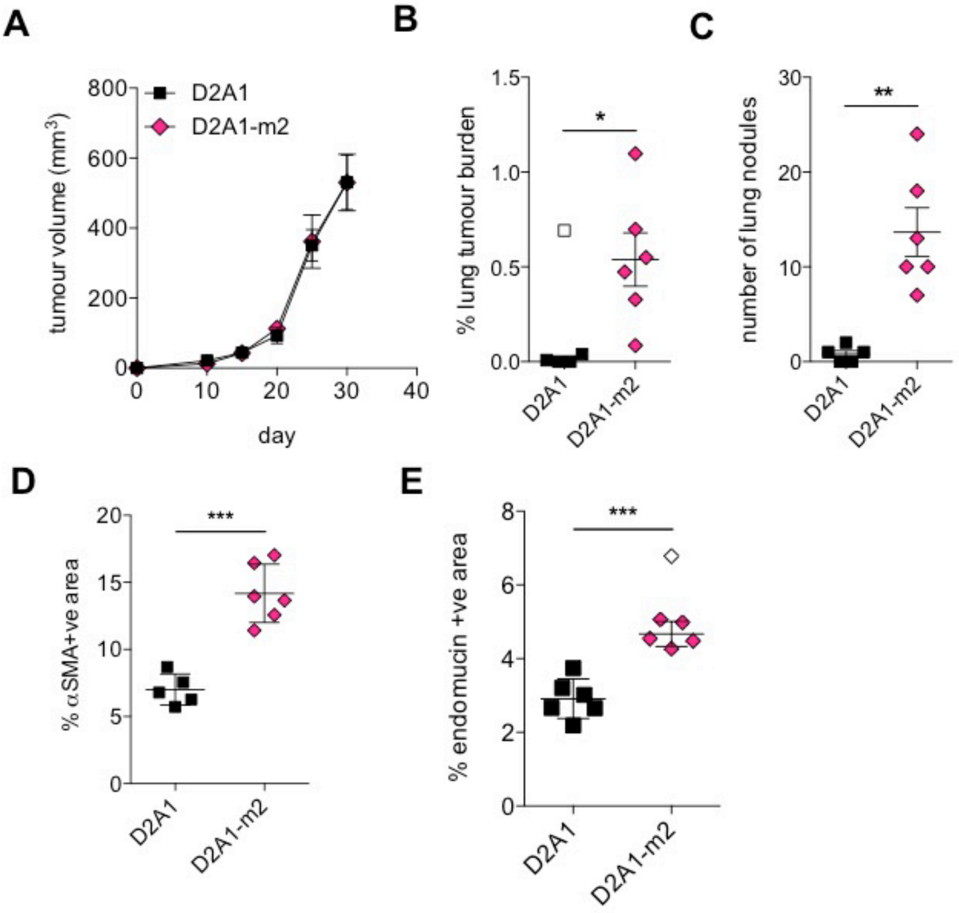

Supplementary Figure S1 Spontaneous metastasis assay in BALB/c mice. 5 x 10^4 D2A1 or D2A1-m2 cells were inoculated into the 4th mammary fat pad of BALB/c mice (n = 5 or 6 mice per group) and culled on day 30. **A** Primary tumour growth. **B,C** Spontaneous metastasis to the lung assessed by % tumour burden and number of metastatic nodules per lung section. Data shown are mean values per mouse +/- SEM. **D**,**E** Primary tumours were sectioned and stained for **D** the activated fibroblast marker αSMA, or **E** the endothelial marker endomucin. Data shows quantification of staining of 6 tumours per group ±s.e.m. (n > 6 fields of view per tumour).

Disease Models & Mechanisms • Supplementary information

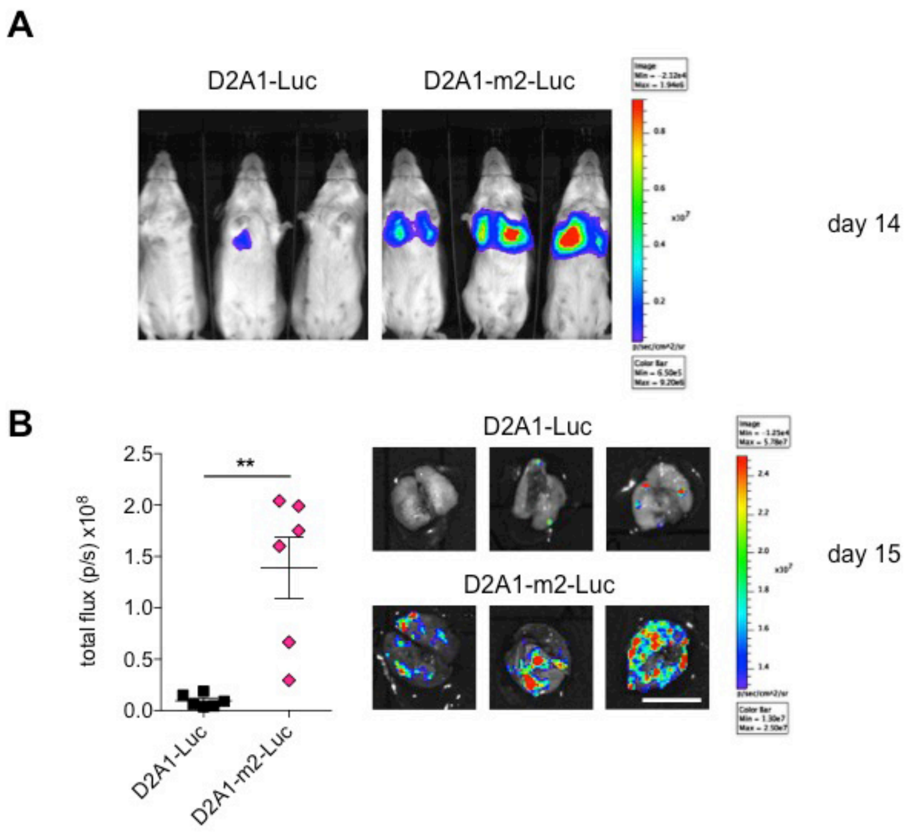

Supplementary Figure S2 Experimental lung metastasis assay with luciferase tagged cells. 4 x 10^5 D2A1-Luc or D2A1-m2-Luc cells were inoculated intravenously into BALB/c mice (n = 6 mice per group). **A** Representative *in vivo* IVIS imaging on day 14. **B** Mice were culled on day 15 when the first mouse showed signs of ill health. Quantification of tumour burden in the lungs via *ex vivo* IVIS imaging. Mean values per mouse ±s.e.m.. Representative *ex vivo* lung IVIS images are shown. Scale bar, 1 cm.

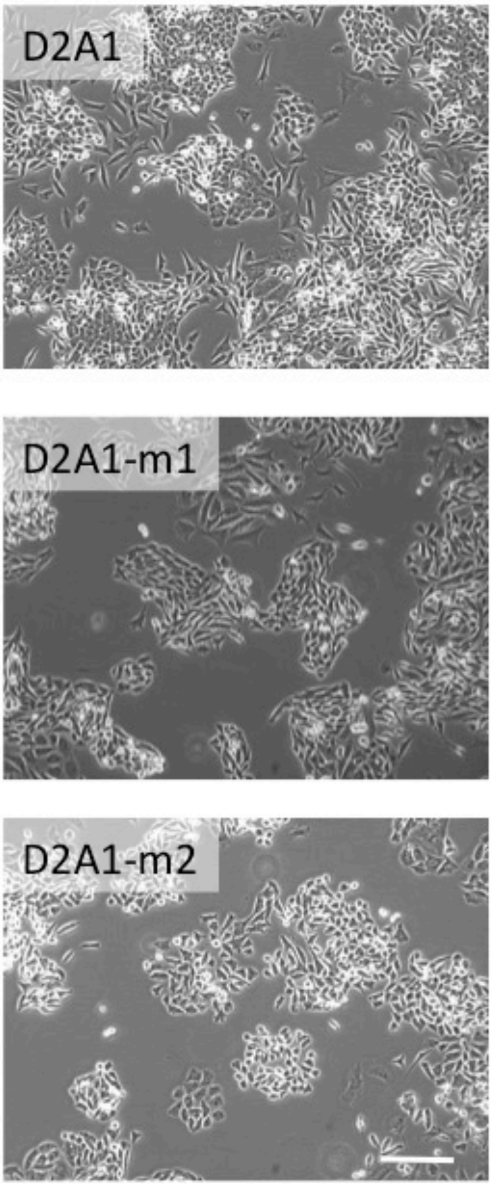

Supplementary Figure S3 Phase contrast images of D2A1, D2A1-m1 and D2A1-m2 cells grown on tissue culture plastic. Scale bar, 400 μm.

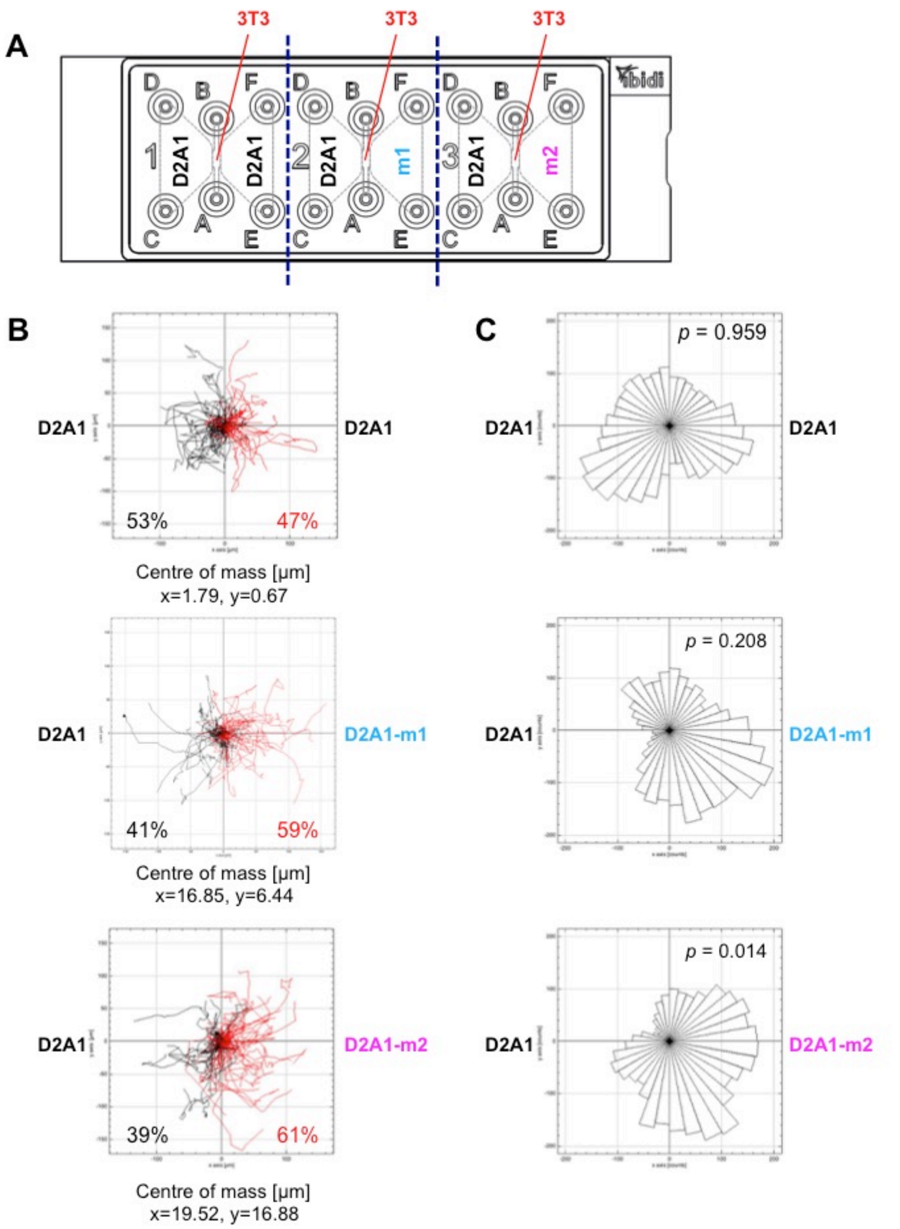

Supplementary Figure S4 Competitive fibroblasts migration assay. **A** Diagram of the experimental setup for the 'ibidi' µ-slide chemotaxis system. Following the manufacturer's instructions, fibroblasts were injected into the central viewing chamber via port A. 3 hours later, D2A1 cells were injected into the left reservoir via port C and either D2A1, D2A1-m1 or D2A1-m2 cells were injected into the right reservoir via port E. Fibroblast migration in the central chamber was imaged over 8 hours (see Methods for further details). **B** Paths taken by individual 3T3 fibroblasts, where all initial starting positions (t=0) are plotted at the origin (x=0, y=0). Red indicates paths with a positive net displacement towards tumour cells seeded on the right hand side. Black paths indicate net displacement towards the left. The percentage of cells exhibiting movement to the left (black) or right (red) is indicated and shown graphically in Fig. 6C, left panel. Coordinates of centre of mass are given below the panels. **C** Corresponding circular histograms, with *p*-values calculated using the Rayleigh test for vector data. Only D2A1-m2 cells induce a significant inhomogeneous distribution of fibroblast trajectories.

Disease Models & Mechanisms • Supplementary information

Supplementary Table S1. Top 40 genes differentially expressed in D2A1-m1 cells *vs.* D2A1 cells

Symbol	Gene name	Accession Number	Fold change	Parametric *p*-value
Mgp	Matrix Gla protein	NM_008597	16.95	< 1e-07
Arhgdib	Rho, GDP dissociation inhibitor beta	NM_007486	13.33	5.10E-05
Fgf7	Fibroblast Growth Factor 7	NM_008008	12.35	1.71E-05
Itgbl1	Integrin Subunit Beta Like 1	NM_145467	6.67	2.21E-05
Thbs2	Thrombospondin 2	NM_011581	6.25	1.30E-06
Pdgfrl	Platelet-derived growth factor receptor-like	NM_026840	5.88	2.70E-06
Id4	Inhibitor of DNA binding 4	NM_031166	5.88	7.40E-06
Tnfrsf11b	TNF receptor superfamily member 11b	NM_008764	5.56	2.93E-05
Lgals7	Galectin 7	NM_008496	5.56	3.59E-05
Akr1c18	Aldo-keto reductase family 1, member C18	NM_134066	5.00	2.40E-06
Ly6a	Lymphocyte antigen 6 complex, locus A	NM_010738	5.00	4.80E-06
Tpm2	Tropomyosin 2, beta	NM_009416	5.00	1.10E-05
Chst1	Carbohydrate (keratan sulfate Gal-6) sulfotransferase 1	NM_023850	4.17	5.00E-07
Ifi27	Interferon, alpha-inducible protein 27	NM_029803	4.17	8.64E-04
AA467197		NM_001004174	4.00	3.00E-07
Casp1	Caspase 1	NM_009807	4.00	6.88E-04
Aqp1	Aquaporin 1	NM_007472	3.45	5.17E-05
Krt8	Keratin 8	NM_031170	3.45	1.19E-04
Ptrf	Caveolae associated 1	NM_008986	3.23	2.70E-05
Vcam1	Vascular cell adhesion molecule 1	NM_011693	3.23	4.65E-04
Aqp5	Aquaporin 5	NM_009701	3.13	4.00E-07
Ank	Progressive ankylosis	NM_020332	3.03	1.00E-07
Idb4	Inhibitor of DNA binding 4	AK041164	3.03	7.50E-06
Aldh3a1	Aldehyde dehydrogenase 3 family member A1	NM_007436	3.03	4.91E-05
Lxn	Latexin	NM_016753	2.94	3.20E-06
D14Ertd668e	PHD finger protein 11D	NM_199015	2.70	5.70E-06
Tmem86a	Transmembrane protein 86A	NM_026436	2.70	1.77E-05
LOC100046616		XM_001476512	2.63	2.00E-07
AI467606		NM_178901	2.63	1.28E-05
Ecm1	Extracellular matrix protein 1	NM_007899	2.63	1.61E-04
Cgn	Cingulin	XM_001001375	-2.59	1.10E-06
Tuft1	Tuftelin 1	NM_011656	-2.59	4.90E-05
Ak3	Adenylate kinase 3	NM_021299	-2.87	1.61E-04
Bgn	Biglycan	NM_007542	-2.98	3.05E-05
1810015A11Rik	YdjC homolog	NM_026940	-3.01	5.45E-05
Ogn	Osteoglycin	NM_008760	-3.10	5.62E-05
Tpd52	Tumor protein D52	NM_009412	-3.15	5.40E-06
Chchd10	Coiled-coil-helix-coiled-coil-helix domain containing 10	NM_175329	-3.52	4.25E-04
4732462B05Rik		AK028848	-4.13	1.85E-05
Ptx3	Pentraxin related gene	NM_008987	-4.85	3.93E-05

Disease Models & Mechanisms • Supplementary information

Supplementary Table S2. Top 40 genes differentially expressed in D2A1-m2 cells *vs.* D2A1 cells

Symbol	Gene name	Accession number	Fold change	Parametric *p*-value
Casp1	Caspase 1	NM_009807	10.99	4.30E-06
Arhgdib	Rho, GDP dissociation inhibitor beta	NM_007486	10.99	1.22E-05
Lgals7	Galectin 7	NM_008496	9.09	7.00E-07
Fgf7	Fibroblast Growth Factor 7	NM_008008	7.69	6.40E-06
Itgbl1	Integrin Subunit Beta Like 1	NM_145467	5.00	6.20E-06
Tpm2	Tropomyosin 2, beta	NM_009416	4.76	6.50E-06
Mgp	Matrix Gla protein	NM_008597	4.55	5.50E-06
Tnfrsf11b	TNF receptor superfamily member 11b	NM_008764	4.17	1.45E-05
Krt8	Keratin 8	NM_031170	3.70	5.90E-06
Vcam1	Vascular cell adhesion molecule 1	NM_011693	3.57	4.64E-05
Id4	Inhibitor of DNA binding 4	NM_031166	3.45	3.70E-06
Thbs2	Thrombospondin 2	NM_011581	3.45	1.31E-05
Pdgfrl	Platelet-derived growth factor receptor-like	NM_026840	3.03	6.40E-06
Ptrf	Caveolae associated 1	NM_008986	2.86	2.80E-06
Gadd45g	Growth arrest and DNA-damage-inducible 45 gamma	NM_011817	2.78	7.21E-05
Rab32	RAB32, member RAS oncogene family	NM_026405	2.70	8.90E-06
Lxn	Latexin	NM_016753	2.63	9.00E-07
Csrp2	Cysteine and glycine-rich protein 2	NM_007792	2.56	1.10E-06
Eno3	Enolase 3	NM_007933	2.56	3.00E-06
Sparc	Secreted acidic cysteine rich glycoprotein	NM_009242	2.56	6.02E-05
Id2	Inhibitor of DNA binding 2	NM_010496	2.56	1.15E-04
Nuak1	NUAK family, SNF1-like kinase, 1	NM_001004363	2.50	1.40E-06
Tmem86a	transmembrane protein 86A	NM_026436	2.44	3.50E-06
Dap	death-associated protein	NM_146057	2.33	4.40E-05
AA467197		NM_001004174	2.33	6.03E-05
Gpnmb	Glycoprotein (transmembrane) nmb	NM_053110	2.33	5.42E-04
AI467606		NM_178901	2.27	1.40E-06
Akr1c18	Aldo-keto reductase family 1, member C18	NM_134066	2.27	1.59E-04
Csnk	Casein kappa	NM_007786	-2.28	1.63E-04
Sox12	SRY (sex determining region Y)-box 12	NM_011438	-2.32	6.10E-06
2810003C17 Rik	Allograft inflammatory factor 1-like	NM_145144	-2.32	1.38E-05
Slpi	Secretory leukocyte peptidase inhibitor	NM_011414	-2.36	2.79E-04
Wfdc2	WAP four-disulfide core domain 2	NM_026323	-2.63	3.76E-05
1810015A11 Rik	YdjC homolog	NM_026940	-2.69	1.23E-05
Chchd10	Coiled-coil-helix-coiled-coil-helix domain containing 10	NM_175329	-2.74	3.09E-04
Bgn	Biglycan	NM_007542	-2.83	2.00E-07
Olfml2b	Olfactomedin-like 2B	NM_177068	-2.87	7.26E-05
LOC100048 733		XM_001481081	-3.07	1.50E-06
Ak3	Adenylate kinase 3	NM_021299	-3.27	1.93E-05
4732462B05 Rik		AK028848	-4.13	2.70E-06

Disease Models & Mechanisms • Supplementary information

Supplementary Table S3. Top 40 genes differentially expressed in both D2A1-m1 and D2A1-m2 *vs.* D2A1 cells

Symbol	Gene name	Accession number	Average fold change
Arhgdib	Rho, GDP dissociation inhibitor beta	NM_007486	12.13
Mgp	Matrix Gla protein	NM_008597	10.63
Fgf7	Fibroblast Growth Factor 7	NM_008008	10.10
Casp1	Caspase 1	NM_009807	7.46
Lgals7	Galectin 7	NM_008496	7.25
Itgbl1	Integrin Subunit Beta Like 1	NM_145467	5.88
Tpm2	Tropomyosin 2, beta	NM_009416	4.97
Thbs2	Thrombospondin 2	NM_011581	4.93
Tnfrsf11b	TNF receptor superfamily member 11b	NM_008764	4.89
Id4	Inhibitor of DNA binding 4	NM_031166	4.62
Pdgfrl	Platelet-derived growth factor receptor-like	NM_026840	4.54
Akr1c18	Aldo-keto reductase family 1, member C18	NM_134066	3.58
Krt8	Keratin 8	NM_031170	3.58
Vcam1	Vascular cell adhesion molecule 1	NM_011693	3.40
AA467197		NM_001004174	3.20
Ptrf	Caveolae associated 1	NM_008986	3.03
Chst1	Carbohydrate (keratan sulfate Gal-6) sulfotransferase 1	NM_023850	3.00
Lxn	Latexin	NM_016753	2.78
Aqp1	Aquaporin 1	NM_007472	2.76
Idb4	Inhibitor of DNA binding 4	AK041164	2.59
Tmem86a	Transmembrane protein 86A	NM_026436	2.59
Gadd45g	Growth arrest and DNA-damage-inducible 45 gamma	NM_011817	2.49
Ank	Progressive ankylosis	NM_020332	2.45
AI467606		NM_178901	2.44
Dap	death-associated protein	NM_146057	2.44
Aqp5	Aquaporin 5	NM_009701	2.38
Axl	AXL receptor tyrosine kinase	NM_009465	2.27
Ecm1	Extracellular matrix protein 1	NM_007899	2.25
Gli2	GLI-Kruppel family member GLI2	NM_001081125	-2.31
Tuft1	Tuftelin 1	NM_011656	-2.37
Csnk	Casein kappa	NM_007786	-2.39
Cgn	Cingulin	XM_001001375	-2.41
Ogn	Osteoglycin	NM_008760	-2.67
LOC100048733		XM_001481081	-2.81
1810015A11Rik	YdjC homolog	NM_026940	-2.85
Bgn	Biglycan	NM_007542	-2.91
Ak3	Adenylate kinase 3	NM_021299	-3.07
Chchd10	Coiled-coil-helix-coiled-coil-helix domain containing 10	NM_175329	-3.13
Ptx3	Pentraxin related gene	NM_008987	-3.46
4732462B05Rik		AK028848	-4.13

Supplementary Table S4. Top cellular and molecular functions identified by IPA

	p-value	Activation z-score	# genes
**D2A1-m1 *vs.* D2A1**			
Cellular Movement	3.63E-03 - 5.69E-09		79
migration of cells*	5.69E-09	2.105	67
migration of breast cancer cell lines*	4.28E-05	2.179	14
Cell Death and Survival	3.49E-03 - 7.79E-08		101
cell survival*	2.83E-03	2.012	38
Cellular Assembly and Organisation	3.19E-03 - 6.74E-07		65
organization of cytoplasm*	6.74E-07	2.355	52
organization of cytoskeleton*	8.45E-06	2.567	46
Cellular Function and Maintenance	3.77E-03 - 6.74E-07		103
Cellular Development	3.50E-03 - 1.03E-06		92
**D2A1-m2 *vs.* D2A1**			
Cellular Movement	2.19E-03 - 1.54E-11		82
cell movement*	1.54E-11	2.414	79
migration of cells*	4.53E-11	2.602	72
Cell Death and Survival	1.73E-03 - 1.70E-08		105
cell death of connective tissue cells*	5.10E-04	2.113	21
cell survival*	8.36E-04	2.535	40
cell viability*	1.73E-03	2.604	37
Cellular Assembly and Organisation	2.04E-03 - 4.56E-08		76
Cellular Function and Maintenance	1.93E-03 - 4.56E-08		94
Cellular Development	2.22E-03 - 7.64E-08		102
**Common in D2A1-m1 and D2A1-m2 *vs.* D2A1**			
Cellular Movement	5.76E-03 - 7.79E-07		41
migration of cells*	7.49E-07	2.125	21
Cell Morphology	5.62E-03 - 7.49E-05		21
Cellular Assembly and Organisation	5.62E-03 - 7.49E-05		32
Small Molecule Biochemistry	5.62E-03 - 9.36E-05		16
Cell-To-Cell Signaling and Interaction	5.62E-03 - 1.05E-04		24
binding of connective tissue cells*	1.16E-03	2.394	6
adhesion of connective tissue cells*	4.13E-03	2.198	5

*Predicted activation, z-score >2 or inactivation, z-score <-2

Supplementary Table S5. Top upstream regulators identified by IPA

	p-value of overlap	Activation z-score
**D2A1-m1 *vs.* D2A1**		
TP53	7.17E-13	1.962
TGFB1	2.44E-12	0.532
HRAS	8.46E-11	1.551
CTNNB1	1.20E-10	1.281
ERBB2	3.15E-10	0.665
KRAS	9.12E-10	0.561
IKBKB	2.01E-08	1.227
**HTT***	**2.25E-08**	**2.128**
MYC	2.28E-08	-0.576
LH	5.19E-08	1.567
**D2A1-m2 *vs.* D2A1**		
**TGFB1***	**8.58E-19**	**2.651**
TP53	2.95E-12	1.681
MYC	6.61E-11	0.869
TNF	3.91E-09	1.962
TGFBR2	4.28E-09	1.083
**SMARCA4***	**9.13E-09**	**2.159**
CTNNB1	7.10E-08	1.610
IKBKB	8.30E-08	-0.077
HRAS	8.39E-08	0.597
KRAS	2.52E-07	0.077
**Common in D2A1-m1 and D2A1-m2 *vs.* D2A1**		
TGFB1	1.50E-11	1.129
KRAS	1.87E-08	0.218
IKBKB	3.43E-08	0.705
CTNNB1	5.50E-07	1.370
NFKBIA	1.02E-06	-1.021
MYC	2.58E-06	0.036
SMO	4.62E-06	
HRAS	5.24E-06	0.186
SMARCA4	7.66E-06	1.718
TP53	1.06E-05	1.176

*Predicted activation, z-score >2 or inactivation, z-score <-2

Disease Models & Mechanisms • Supplementary information
